# How Does the Spatial Confinement of FtsZ to a Membrane Surface Affect Its Polymerization Properties and Function?

**DOI:** 10.3389/fmicb.2022.757711

**Published:** 2022-05-03

**Authors:** Marisela Vélez

**Affiliations:** Instituto de Catálisis y Petroleoquímica, Consejo Superior de Investigaciones Científicas (CSIC), Madrid, Spain

**Keywords:** bacterial division, bacterial cytoskeletal proteins, FtsZ, lipid membrane, polymerization

## Abstract

FtsZ is the cytoskeletal protein that organizes the formation of the septal ring and orchestrates bacterial cell division. Its association to the membrane is essential for its function. In this mini-review I will address the question of how this association can interfere with the structure and dynamic properties of the filaments and argue that its dynamics could also remodel the underlying lipid membrane through its activity. Thus, lipid rearrangement might need to be considered when trying to understand FtsZ’s function. This new element could help understand how FtsZ assembly coordinates positioning and recruitment of the proteins forming the septal ring inside the cell with the activity of the machinery involved in peptidoglycan synthesis located in the periplasmic space.

## Introduction

Bacterial division requires a profound morphological change in the cell. It involves the concerted action in space and time of different cellular elements: chromosomes divide and distribute between the new cells as proteins forming the divisome localize in the center and promote active membrane deformation and peptidoglycan synthesis required to separate the cell into two new ones (Mahone and Goley, 2020).

The study of this fascinating field is considered to have started with the first description of the FtsZ ring in 1991 made by Bi and Lutkenhaus (1991). Since then, we have gained huge insight into how this complex process takes place. A combination of genetic and biochemical studies *in vitro* and *in vivo* have provided knowledge of the essential proteins participating and how they interact and assemble. There are several reviews summarizing our current knowledge of the formation of the septal ring (Adams and Errington, 2009; Mingorance et al., 2010; Den Blaauwen and Luirink, 2019; Du and Lutkenhaus, 2019; Mahone and Goley, 2020; McQuillen and Xiao, 2020; Barrows and Goley, 2021). Advances in single molecule fluorescence, applied both to reconstituted systems (Caldas et al., 2019; García-Soriano et al., 2020) and to whole cells (McCausland et al., 2021; Squyres et al., 2021; Yang et al., 2021), have allowed a more detailed analysis of the structural rearrangements occurring during this active energy-consuming process. These studies have revealed a highly dynamic and concerted interaction between septal ring and peptidoglycan synthesis proteins (Bisson-Filho et al., 2017; Yang et al., 2017; McCausland et al., 2021; Squyres et al., 2021).

FtsZ is the cytoskeletal protein that organizes the formation of the septal ring and orchestrates the division process (Margolin, 2005; Huang et al., 2013; Ortiz et al., 2015; Barrows and Goley, 2021). Since its first description (Dai and Lutkenhaus, 1991; Pla et al., 1991; de Boer et al., 1992; RayChaudhuri and Park, 1992), much work has focused in understanding how it behaves *in vitro* and how it interacts with other proteins. We know that membrane association is essential for its function and that, even when attached through an inserted transmembrane region, in the absence of any of the proteins present *in vivo*, it displays a dynamic rich behavior and deforms membranes (Osawa et al., 2009; Caldas et al., 2019; Ramirez-Diaz et al., 2021).

We have identified a large catalog of proteins participating in the formation of the septal ring, but we still have very few cues about how they communicate to coordinate cell division. Information is transferred between proteins located inside the membrane, that actively condense to form the septal ring, and proteins whose function involves communication and transfer of material across the membrane. Peptidoglycan building blocks are exported to the periplasmic space for the peptidoglycan synthetic machinery to work (Ruiz, 2016). Direct protein-protein interactions are frequently evoked as the main element guiding the concerted process, but it is difficult to imagine that the fluid membrane that hosts many of the proteins involved plays no role, as is frequently depicted in illustrations.

Membranes are not passive elements (Kalappurakkal et al., 2020), particularly when associated to active cytoskeletal proteins that form polymeric filamentous structures that reorganize by dissipating energy consumed from ATP or GTP hydrolysis (Litschel et al., 2021). The long-range order of cytoskeletal filaments is ideal for building sufficiently large structures responsive to multiple inputs that can generate mechanical forces. It is therefore not surprising that several bacterial cytoskeletal proteins play essential roles in cell division (Shih and Rothfield, 2006; Cabeen and Jacobs-Wagner, 2010; Ingerson-Mahar and Gitai, 2012; Pilhofer and Jensen, 2013; Busiek and Margolin, 2015; Siliakus et al., 2017). Some are involved in DNA segregation, ParM for example, and others, such as eukaryotic actin and tubulin homologues MreB, FtsA, and FtsZ, are associated to the membrane and play a major role in maintaining cell shape and remodeling the membrane.

Studies in eukaryotes have shown that active cytoskeletal proteins regulate lipid and protein distribution and membrane mechanical properties, originating non-equilibrium distributions and emerging properties (Gowrishankar et al., 2012; Longo, 2018; Steinkühler et al., 2019; Shaw et al., 2020). The actin cortex may influence plasma membrane organization either by direct interaction with lipids and proteins or through the flow of actin filaments generated by myosin-induced stresses. This activity can also drive actin-associated membrane components out of equilibrium inducing active processes such as persistent advection, clustering, and anomalous density fluctuations, even though measurable hydrodynamic flows in the plasma membrane of unconnected components are not present. The remodeling of the actomyosin layer also affects phase-segregation in the membrane bilayer. It has been experimentally shown that actin activity changes the size and dynamics of the formed domains and that, in addition, membrane domains influence the actin organization (Köster et al., 2016). Lipid domains, referred to as rafts, play an important role in regulating functions such as membrane trafficking (Redpath et al., 2020) and response of membrane receptors (Gowrishankar et al., 2012). Other membrane associated GTP self-aggregating proteins such as dynamins (Kalia and Frost, 2019) and septins (Lam and Calvo, 2019), also reshape the lipid membrane in eukaryotic cells by undergoing GTP-induced conformational changes, although the details of how this happens are still to be elucidated (Pannuzzo et al., 2018). Thus, the current picture of a cell membrane, based on experiments mostly carried out in eukaryotes, is that of a composite material in which lipids and proteins interact to transfer information across membrane components (Sezgin et al., 2017).

In this minireview I will focus on FtsZ polymerization on membranes, but I will first briefly update what we know about bacterial membranes to highlight the evidence that indicates that we should extrapolate what we have learned about cytoskeletal membrane associated proteins in eukaryotes to better understand what is happening in the bacterial membrane during cell division.

To what extent is the presence of the membrane surface and the confinement it imposes to the FtsZ filaments relevant to reveal the rich dynamic behavior observed? Can we also expect bacterial membranes to be responsive to the activity of membrane associated cytoskeletal proteins, as has been shown for eukaryotic cytoskeleton? Could protein modulated membrane properties participate in coordinating the complex interactions acting on the cell division machinery? These are the questions I will address. First, I will briefly summarize advances in our understanding of bacterial membrane organization. I will then briefly review what we know about the active and dynamic FtsZ filaments and their interaction with the membrane. In the discussion I will argue that it might be interesting to attend the role the lipid membrane could play in regulating the process in order to fully understand how bacterial cell division occurs.

## The Bacterial Membrane

Bacterial membranes have been less subject to biophysical studies than eukaryotic membranes. Their small size and the complexity rendered by the presence of a peptidoglycan layer make their characterization difficult. However, spatial and temporal reorganization of lipids and proteins on bacterial surfaces take place during complex functions, just as happens in eukaryotes. High resolution optical fluorescence microscopy has confirmed that their inner membrane shares the lateral heterogeneity and complexity found in eukaryotic cells (Dempwolff et al., 2016; Raghunathan and Kenworthy, 2018). Domains and lipid rafts in bacterial membranes are highly organized with specific lipids and associated proteins forming specialized subcellular compartments. They have been referred to as functional membrane microdomains (FMM) to differentiate them from the eukaryotic lipids rafts containing cholesterol, not present in bacteria. The lateral segregation has been observed to be associated to the presence of anionic lipids, squalene, phosphatidyl-ethanolamine, and proteins such as flotillins (Nishibori et al., 2005; Mileykovskaya and Dowhan, 2009; Bramkamp and Lopez, 2015; Lin and Weibel, 2016; Strahl and Errington, 2017; Yokoyama and Matsui, 2020).

Membrane structure and dynamics are interdependent. Structural heterogeneity has consequences in the heterogeneous dynamics of proteins on the membrane (Nenninger et al., 2014; Adhyapak et al., 2020; Pluhackova and Horner, 2021). Therefore, membrane composition also plays a regulatory role in cell physiology. Stress is known to trigger metabolic pathways that sense threats and mount a protective response involving modification of cell wall composition through the regulation of biosynthetic pathways of their components (Rowlett et al., 2017; Willdigg and Helmann, 2021). There is evidence that certain lipids are enriched in the division site (Mileykovskaya and Dowhan, 2005), indicating that the division process is no exception.

In summary, experimental results indicate that non-random distribution of proteins and lipids also play an important role in protein-protein interactions in bacteria (Raghunathan and Kenworthy, 2018; Zeno et al., 2020), as is well established for eukaryotic membranes.

## FtsZ

### Conformation and Dynamics in Solution

**FIGURE 1:**
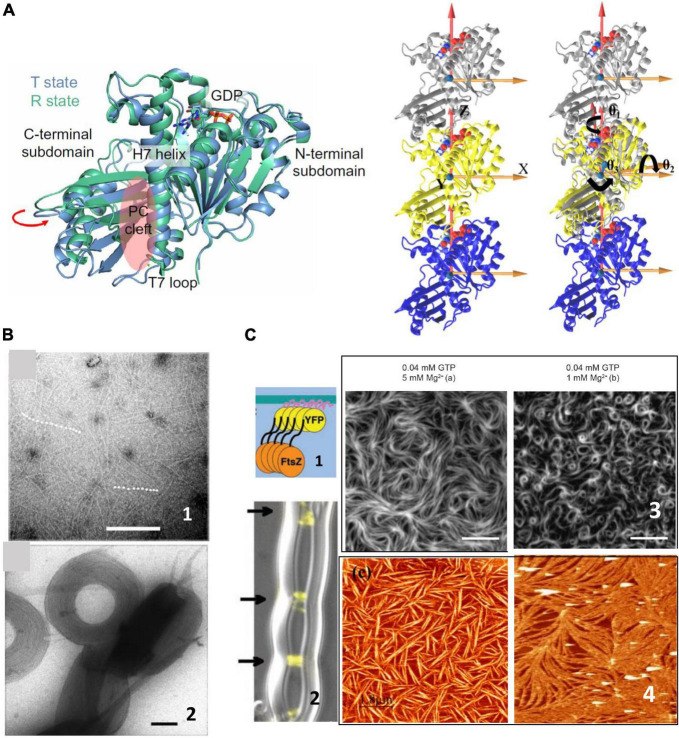
FtsZ structure and polymerization in solution. **(A)** Left shows the two crystal structures of FtsZ from Staphylococcus aureus corresponding to the T and R state conformations in the same crystal, indicating the structural equilibrium of the two states (adapted from Fujita et al., 2017). The trimers on the right show the definitions of bending and twisting angles obtained from molecular dynamics simulations. The left trimer shows the coordinate system and the one on the right shows the bending angles, θ_1_ (rotations around the Z axis), θ_2_ (rotations around the X axis), and θ_3_ (rotations around the Y axis), tracked by calculating the rotations of the top (silver) subunit to align the initial reference frame of the middle subunit (adapted from Lv et al., 2021). **(B)** Shows representative negative stained TEM images of FtsZ filaments in solution (1) and aggregates formed in the presence of crowding agents (2). Scale bar is 100 nm (adapted from Popp et al., 2009). **(C)** Shows surface confined FtsZ polymers. (1) Show a chimeric FtsZ containing a fluorescent protein and a membrane targeting sequence (MTS) on liposomes (2) (adapted from Osawa et al., 2008) and on planar supported membranes (3) (adapted from Ramirez-Diaz García-Soriano et al., 2018). Scale bars are 5 μm. (4) Shows Atomic Force Microscopy images of FtsZ filaments attached covalently to a lipid membrane (left) (adapted from Encinar et al., 2013) or bound through the protein ZipA (right) (adapted from Mateos-Gil et al., 2012a). Scale bars are 2 μm.

FtsZ is a soluble 40.3 kDa protein that binds and hydrolyzes GTP and shows a striking structural similarity to tubulins (Erickson, 1997). Its crystal structure was determined in 1998 (Löwe and Amos, 1998) and since then, much biochemical work on the isolated protein in solution has described its polymerization properties. Lateral interactions between individual filaments participate in bundling (Hörger et al., 2008a; Milam et al., 2012), affect their arrangement on surfaces (Márquez et al., 2017) and are strongly modulated by the presence of different ions or crowding agents (Erickson et al., 1996; Popp et al., 2009; Figure 1). The presence of curvature in the filaments has been controversial. In analogy to tubulin, it was first considered that the presence of GDP induced the curved conformation whereas the GTP loaded monomers formed straight filaments (Lu et al., 2000; Hsin et al., 2012). However, crystal structures of monomers containing GDP and GTP were not able to confirm this association of the nucleotide phosphorylation state with the degree of curvature (Oliva et al., 2004), and conditions in which the monomers contained essentially phosphorylated nucleotide were observed to be highly curved (Mateos-Gil et al., 2012b; Loose and Mitchison, 2013; Ramirez-Diaz García-Soriano et al., 2018). More recent structural characterizations, coming both from structural and molecular dynamics simulations, have depicted highly flexible monomer in which the relative orientation of the C-terminal and N-terminal domains, linked through a helix 7, can change up to nearly 30 degrees (Martín-Galiano et al., 2010; Matsui et al., 2012; Fujita et al., 2017; Wagstaff et al., 2017). Two monomer conformations have been described, open and close, also referred to as relaxed and tense, that appear to be associated to whether the monomer is isolated or polymerized (Schumacher et al., 2020; Figure 1). Molecular dynamics simulations have also revealed the presence of a significant angle between monomers that confers a twist to the filaments (Hsin et al., 2012; Gonzalez de Prado Salas et al., 2014; Lv et al., 2021). Experimental observations have confirmed that filaments on surfaces indeed manifest this twist (Arumugam et al., 2012; Gonzalez de Prado Salas et al., 2014; Ramirez-Diaz et al., 2021).

Early studies showed that FtsZ polymers are dynamic, both *in vivo* and *in vitro* (Stricker et al., 2002; Chen and Erickson, 2005, 2009; Chen et al., 2005), where filaments studied in solution revealed instability due to continuous monomer exchange associated to GTP hydrolysis (Romberg and Levin, 2003). Concentration dependent cooperative polymerization was detected quite early and opened questions regarding how individual filaments, the structures mainly observed at the time, could account for cooperativity, more compatible with the existence of double filaments (Chen et al., 2005; Erickson, 2017). Later cooperativity was attributed to a conformational switch (Huecas and Andreu, 2003) in the monomer that facilitates polymerization, in line with the increasing evidence that monomers are flexible (Martín-Galiano et al., 2010).

All together, the picture that emerged from biochemical and structural studies of filaments forming in solution was of soft, flexible, and polymorphic assemblies that can adopt a large range of shapes easily malleable through environmental conditions (Figure 1). A yet open challenge is to understand how such dynamic structures are responsible for coordinating and maintaining the formation of the septal ring when associated to the membrane.

### Surface Confined Polymer Structure and Dynamics

The dynamic behavior of FtsZ showed unexpected complexity when filaments were observed associated to a surface. Although some biochemical studies in solution gave hints that polymer growth could be directional (Osawa and Erickson, 2005; Redick et al., 2005), it was not until single molecule fluorescence allowed visualizing polymers associated to membranes, both *in vivo* and *in vitro*, that dynamics of FtsZ filaments was clearly observed. Several groups confirmed that inside the cell FtsZ moves along the cell circumference in a treadmilling fashion along the cell-wall remodeling machinery (Bisson-Filho et al., 2017; Yang et al., 2017; Perez et al., 2019). Studies *in vitro*, also showed that FtsZ filaments on supported membranes manifested unexpected cooperative behavior based on treadmilling (Loose and Mitchison, 2013), forming vortices moving in opposite directions depending on the orientation of the monomers on the surface (Ramirez-Diaz García-Soriano et al., 2018). This behavior does not require any additional proteins, only a chimeric FtsZ containing a fluorescent protein and a membrane targeting sequence (MTS) incorporated at one end to allow for membrane attachment (Ramirez-Diaz García-Soriano et al., 2018; Figure 1). Addition of filament binding proteins such as ZapA modulate precision and robustness of the collective behavior (Caldas et al., 2019), but not the essential traits, suggesting that FtsZ itself contains the required information to treadmill and rearrange on surfaces.

Experiments have shown that surface attachment regulates filament dynamics, as had been previously predicted from theoretical models (Mateos-Gil et al., 2019). Removing the C-terminal flexible linker abolishes treadmilling (García-Soriano et al., 2020). Filaments bind to several membrane associated regulatory proteins, such as FtsA, ZipA, or MinC, through the C-terminal constant region located at the end of a non-structured C-terminal linker (Buske and Levin, 2013; Gardner et al., 2013). This disordered region can vary in length between species but is required for proper cell division (Ohashi et al., 2002; Buske et al., 2015; Cohan et al., 2020). One interpretation is that regulating the distance and attachment strength through a flexible spacer is important for modulating the dynamic assembly. In some organisms, FtsZ binds to the membrane through ZipA, which provides an additional non-structured region that sits between the FtsZ monomer and the surface (Ohashi et al., 2002; Mateos-Gil et al., 2016). We can only speculate about why these flexible spacers and distance from the membrane are important for FtsZ function. We do not know if they affect the monomer conformational switch that has been proposed to regulate polymer dynamics. Another possibility is that attachment strength modulates filament twist and thus filament mechanical properties, as has been suggested by theory (Gonzalez de Prado Salas et al., 2014; Mateos-Gil et al., 2019). Modulating the compactness of this variable region through the binding of regulatory proteins could be a way to affect filament dynamics and structure. Filaments grow from both ends, without treadmilling (Márquez et al., 2019), when they are firmly attached to a membrane with a defined orientation, confirming that surface attachment and orientation have a strong impact on their structure and dynamics on membranes.

Surface confinement and lipid type modulate both filament dynamics and shape (Mateos-Gil et al., 2012a; Encinar et al., 2013). Filament’s preferential curvature, lateral interactions and twist (Hörger et al., 2008a,b; Paez A. et al., 2009; Paez P. et al., 2009; Mateos-Gil et al., 2019; Márquez et al., 2019) are revealed differently in solution and under confinement on a two dimensional surface. Theoretical models that include flexible monomer attachment and torsion provide testable hypothesis indicating that only these two elements are enough to induce an asymmetry in the filament ends accessibility to monomer addition that would generate different growth speed on each end, which manifests as treadmilling (Mateos-Gil et al., 2019). Asymmetric confinement to a surface is also likely to regulate the conformation of the flexible monomers and thus polymer dynamics.

### Effect of the FtsZ Polymers Activity on the Lipid Membrane

Filaments on the surface play a GTP-dependent active role on membrane deformation, in spite of their continuous monomer exchange. The debate of whether the force needed to constrict the membrane during cell division comes exclusively from FtsZ filaments or from the peptidoglycan synthetic machinery is still open, although there is no question that at least some membrane deformation is provided by the protein activity (Xiao and Goley, 2016). Filaments create concave or convex deformations or inwards cone structures emerging from the membrane surface (Xiao and Goley, 2016), depending on whether they interact through the N-terminal or the C-terminal end of the protein, indicating drilling-like inward forces (Osawa et al., 2008, 2009). One available hypothesis to explain how flexible, dynamic filaments deform the membrane (Xiao and Goley, 2016) is that torsion provides partly the flexibility found in solution or on surfaces where the attachment is loose. Tight anchoring could stiffen the filaments (Gonzalez de Prado Salas et al., 2014), allowing them to readily deform the membrane and exert force (Mateos-Gil et al., 2019).

One question that is seldom addressed is how the activity of the filaments and the monomer exchange due to GTP hydrolysis affects the structure and integrity of the membrane. FtsZ has a GTP hydrolysis rate comparable to monomer turnover time, and releases the hydrolyzed nucleotide very fast (Romberg and Mitchison, 2004). This rapid release of products implies that the energy from GTP hydrolysis is quickly dissipated, in contrast to microtubules, where it is stored as strain in the polymer (Caplow et al., 1994) and released during microtubule disassembly to perform mechanical work.

**FIGURE 2:**
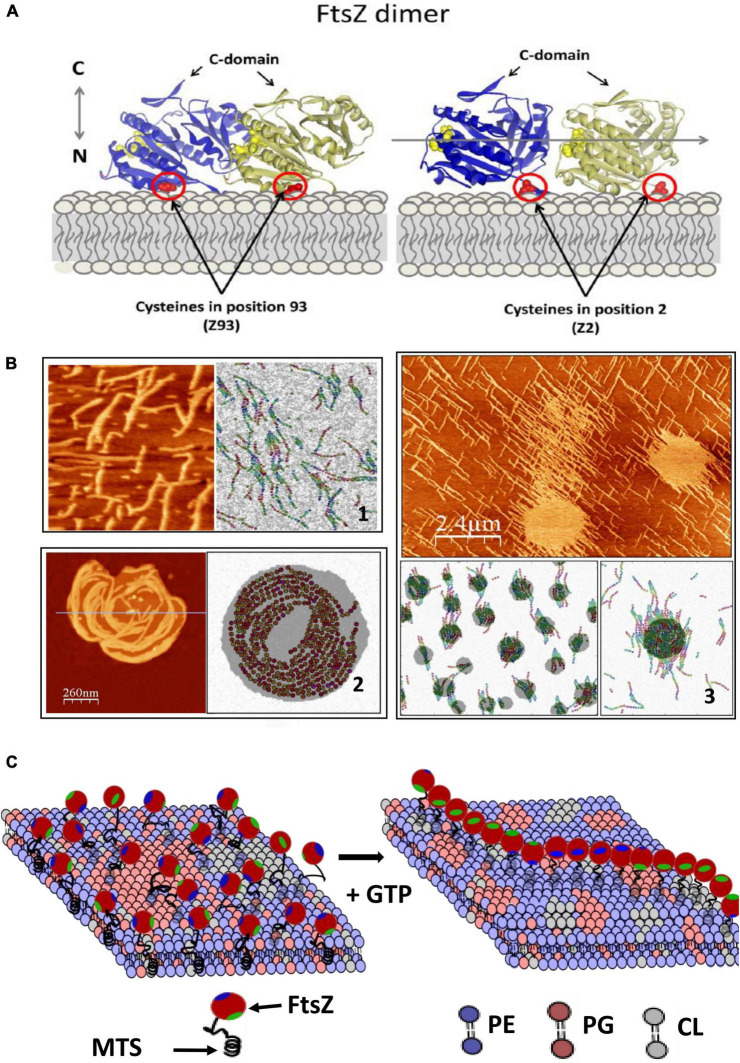
FtsZ activity on the lipid membrane. **(A)** Shows how *E.coli* FtsZ monomers are oriented when attached covalently to a maleimide lipid (DSPE-MAL) in the bilayer through a cysteine placed in different positions. **(B)** Shows 3 examples of structures observed with AFM and snapshots of MC simulations of the model that describes the system including three terms: the dynamic interactions between protein monomers, the interactions between lipid components, and a mixed term considering protein–lipid interactions. Including torsion of the monomers within the filament in the model is necessary to account for the observed filament shapes. (1) Shows mutant E93C above lipid segregation temperature; (2) shows mutant F2C below lipid segregation temperature and (3) mutant E93C at higher DSPE-MAL concentration (adapted from González de Prado Salas et al., 2015). **(C)** Shows a schematic illustration of how polymerization of FtsZ could affect the membrane. The association is through a membrane targeting sequence (MTS) localized in the C-terminal region (green). The components are the main lipids present in the *E.coli* inner membrane (phosphatidylethanolamine, PE, phsophatidylglycerol, PG and cardiolipin, CL). Tension created by filament torsion and preferential curvature restrictions upon surface confinement could affect lipid reorganization.

How does this dissipated energy affect the membrane? There is experimental evidence that FtsZ filaments attached to a supported lipid bilayer affect the shape of lipid domains and that the segregated lipids also influence the distribution and shape of the filaments (González de Prado Salas et al., 2015). Modeling this behavior requires considering filament torsion, monomer exchange, lateral interactions, and preferential curvature (Gonzalez de Prado Salas et al., 2014), and the tendency of the lipids to segregate into domains (Figure 2). Given the complex composition of the lipids of the bacterial inner membrane, it is not difficult to imagine that this lipid-protein interplay will be relevant also in the living cell and that the membrane components will respond to the remodeling of the filaments on its surface. Experiments in model membranes provide evidence that this is indeed the case. Langmuir monolayers of *E. coli* lipids indicate that this lipid mixture is easily stretchable (López-Montero et al., 2008) and that the presence of active FtsZ filaments attached through ZipA further softened the membrane (López-Montero et al., 2012, 2013). The energy dissipated by GTP hydrolysis could be transmitted to the membrane affecting the distribution and size of lipid domains in the inner side of the *E. coli* membrane (Zerrouk et al., 2008). This domain reorganization could affect membrane plasticity and modulate its local mechanical properties (López-Montero et al., 2010). Figure 2C shows a cartoon of how the tension created by filament formation could affect lipid domain redistribution on the membrane.

## Discussion

Condensation of FtsZ filaments in the central position of the inner cell membrane orchestrates the complex process of cell division. This event transmits information along and across the membrane surface through its interaction with many other proteins located both in the membrane as well as protruding from either side, toward the periplasmic space or toward the intracellular space. The aim of this mini-review has been to try to stitch together information that has been gathered from different approaches to the study of FtsZ polymers. One of the important challenges that remains in the field is closing the gap between the information provided by various experimental approaches that access different spatial and temporal scales. Whereas molecular dynamics simulations describe a highly flexible monomer, it is difficult to associate these conformational switches to the cooperative polymerization and treadmilling assembly observed at surfaces. We do not know either how the functionally important C-terminal non-structured region is modulating monomer-monomer interactions or their association to membrane bound anchoring proteins. Individual monomer restructuring undergone during the nucleotide hydrolysis cycle could be affected by surface confinement on a charged lipid surface. However, it is experimentally difficult to associate monomeric conformations to the collective behavior of the filaments observed on the membrane. In order to reconcile all the information available we will most likely need to incorporate additional techniques such as solid state NMR or other spectroscopic techniques to look in more detail at the structural rearrangement of the proteins and the lipids during the ring formation process.

Another issue that I have stressed is the potential importance of the lipid rearrangement due to the activity of FtsZ. There is another example of a membrane associated cytoskeletal protein participating in bacterial division for which there is evidence that its function is associated to membrane properties. MreB is a membrane associated actin homolog required in rod-shaped bacteria for cell shape maintenance and for peptidoglycan synthesis (Dempwolff et al., 2011; Nurse and Marians, 2013; Lee et al., 2020). Its direct interaction with FtsZ is required for septum synthesis and cell division in Escherichia coli (Varma and Young, 2009; Fenton and Gerdes, 2013). It has recently been shown that phospholipid composition and membrane fluidity affect the localization of MreB, which in turn affects peptidoglycan synthesis (Kurita et al., 2020). It has also been shown that MreB promotes membrane fluidity and affects membrane protein localization (Strahl et al., 2014; Oswald et al., 2016). This further illustrates that local modulation of membrane properties might be a strategy to convey information between different proteins involved in the various functions active during cell division.

## Conclusion and Perspectives

There is enough accumulated evidence indicating that proteins and lipids in membranes participate in a concerted fashion to generate complex functions. Active energy consuming proteins near a membrane invest part of this energy to transform membrane structure and properties. Although most of the experimental evidence of these interactions comes from studies done in eukaryotic cells, observations of FtsZ on lipid membranes indicate that in prokaryotic cells this two-way communication between protein activity and membrane properties also exists. Considering the mutual influence between the collective properties of active proteins and membrane lipids offers a wider conceptual framework to understand the intricate regulation of different protein activities during cell division. It is likely to provide additional elements to understand this complex yet fascinating process.

## Author Contributions

MV wrote the article.

## Conflict of Interest

The author declares that the research was conducted in the absence of any commercial or financial relationships that could be construed as a potential conflict of interest.

## Publisher’s Note

All claims expressed in this article are solely those of the authors and do not necessarily represent those of their affiliated organizations, or those of the publisher, the editors and the reviewers. Any product that may be evaluated in this article, or claim that may be made by its manufacturer, is not guaranteed or endorsed by the publisher.
